# A Metabolomic Profile of Seminal Fluid in Extremely Severe Oligozoopermia Suggesting an Epididymal Involvement

**DOI:** 10.3390/metabo12121266

**Published:** 2022-12-15

**Authors:** Orianne Serri, Magalie Boguenet, Juan Manuel Chao de la Barca, Pierre-Emmanuel Bouet, Hady El Hachem, Odile Blanchet, Pascal Reynier, Pascale May-Panloup

**Affiliations:** 1 Reproductive Biology Unit, Angers University Hospital, 49000 Angers, France; 2 MITOVASC, INSERM 1083-CNRS 6015, SFR ICAT, Angers University, IBS–CHU, 49000 Angers, France; 3 Department of Biochemistry and Genetics, Angers University Hospital, 49000 Angers, France; 4 Department of Reproductive Medicine, Angers University Hospital, 49000 Angers, France; 5 Department of Obstetrics and Gynecology, Lebanese American University, Beirut 1100, Lebanon; 6 CRB, BB-0033-00038, Angers University Hospital, 49000 Angers, France

**Keywords:** metabolomics, male infertility, azoospermia, seminal fluid, testicular dysfunction, epididymis

## Abstract

Male infertility has increased in the last decade. Pathophysiologic mechanisms behind extreme oligospermia (EO) are not yet fully understood. In new “omics” approaches, metabolomic can offer new information and help elucidate these mechanisms. We performed a metabolomics study of the seminal fluid (SF) in order to understand the mechanisms implicated in EO. We realized a targeted quantitative analysis using high performance liquid chromatography and mass spectrometry to compare the SF metabolomic profile of 19 men with EO with that of 22 men with a history of vasectomy (V) and 20 men with normal semen parameters (C). A total of 114 metabolites were identified. We obtained a multivariate OPLS-DA model discriminating the three groups. Signatures show significantly higher levels of amino acids and polyamines in C group. The sum of polyunsaturated fatty acids and free carnitine progressively decrease between the three groups (C > EO > V) and sphingomyelins are significantly lower in V group. Our signature characterizing EO includes metabolites already linked to infertility in previous studies. The similarities between the signatures of the EO and V groups are clear evidence of epididymal dysfunction in the case of testicular damage. This study shows the complexity of the metabolomic dysfunction occurring in the SF of EO men and underlines the importance of metabolomics in understanding male infertility.

## 1. Introduction

Recent years have seen an increase in the incidence of male factor infertility in France [1] and all over the world, with a notable decline in the sperm concentration of 1.4 to 1.6% per year, according to the geographical regions [2].

The diagnosis of male factor infertility is based on abnormalities in one or several of the objective sperm parameters. Oligospermia, which is characterized by a decrease in the sperm concentration, is among the most frequently encountered. It is considered mild when the sperm concentration is between 10 and 15 million/mL (M/mL), moderate if the concentration is between 5 and 10 M/mL, and severe when the concentration is lower than 5 M/mL [3]. Extreme oligospermia (EO) are defined by a sperm concentration in the ejaculate lower than 1 M/mL [4,5]. Among these, the most severe is non-obstructive azoospermia (NOA), which is characterized by the complete absence of sperm cells in the ejaculate, confirmed on two separate sperm samples. On the other hand, cryptozoospermia is defined by the absence of sperm cells on the direct examination of fresh preparation, but the presence of a very low number in the pellet after centrifugation [6]. Despite these two pathologies being separate entities, the distinction is not always straightforward [7] and there seems to be a continuum between the different disorders among EO. Indeed, studies have shown that around 20% of men with severe oligoasthenospermia will become crypto/azoospermic in a mean time of 3 years [3,8].

EO is frequently diagnosed during an infertility workup and requires specific management. Indeed, given the high intra-individual variability in sperm parameters, and the risk of progression towards azoospermia, these cases require the cryopreservation of several samples of sperm, and/or testicular biopsies, as part of the preparation for assisted reproductive technologies (ART). On the other hand, most cases of EO are idiopathic, even though genome studies increasingly show underlying genetic causes [9].

Recently, omics technologies, and most notably metabolomics, are being applied in the field of male infertility in an effort to understand its pathophysiology, with the seminal fluid (SF) being at the center of analyses. Several studies have shown that, in patients with oligoasthenospermia (OA), there is an alteration of the energetic metabolism—more specifically the beta-oxidation [10] and glycolysis [11,12,13] pathways—as well as a distortion of the oxidative balance due to a decrease in certain amino acids or biogenic amines [10,14,15], and an alteration of the seminal fluid composition [10,15]. Studies have also confirmed a correlation between the concentrations of certain metabolites and sperm parameters: carnitine and carnosine were linked to concentration [16,17] and fructose linked to mobility [13]. Despite showing interesting results, most of these studies were limited by small samples sizes, the use of multiple techniques targeting different metabolites, and the inclusion of heterogenous populations with varying alterations of sperm parameters. To the best of our knowledge, no study in the literature reported on the use of metabolomics in the seminal fluid to try and find a specific signature that would help understand the pathophysiologic mechanisms of EO. Based on that, we decided to analyze the metabolomic profile of the SF of patients with EO, and compare it with two control groups, one with normal semen parameters according to the WHO criteria, and one with a history of vasectomy. The latter group would allow to differentiate and separately analyze the metabolites in epididymal secretions.

## 2. Materials and Methods

### 2.1. Study Population

We performed a prospective observational study at the Reproductive Medicine Department of the Angers University between October 2018 and February 2021.

We included three groups of patients. The EO group included patients with extreme oligospermia, defined as < 1 M/mL, who presented to our department for an IVF/ICSI treatment, for the cryopreservation of sperm, or for semen analysis. The vasectomy (V) group included patients who presented to our center for a semen analysis ordered by their physician to confirm azoospermia following vasectomy surgery. The control (C) group included patients with normal semen parameters, according to the WHO criteria, whose partners were undergoing intrauterine inseminations (IUI) or IVF/ICSI for female factor infertility.

All patients in the EO group underwent a full urologic workup (physical examination, testicular ultrasound, and genetic testing) in order to rule out obstructive causes. Excluded from the group were all patients with a positive history suggestive of secondary causes of EO, such as orchiepididymitis, hypogonadotropic hypogonadism, or gonadotoxic treatment. 

All patients who were followed at our department during the study period and who signed the consent form were eligible for inclusion. We excluded from the study all patients who refused to sign the consent form, patients who did not fit in any of the three study groups, and patients with associated medical conditions or under chronic medication. None of the included patients had any metabolic condition or had lifestyle habits associated with altered sperm parameters, and none were taking any medications or supplements to improve sperm quality.

### 2.2. Ethical Approval

All participants signed the consent form prior to inclusion, and the study was approved by the Ethics Committee of the University Hospital of Angers, France (Number DC-2014-2224 and AC-2017-2993).

### 2.3. Sample Preparation

All semen samples were collected by masturbation in the andrology laboratory, following an abstinence period of 3 to 5 days. A fraction of the sample was taken for the assessment of the sperm parameters and the planned ART, and the remainder taken for our study. The samples were centrifuged on a density gradient to isolate the seminal plasma, which was later centrifuged for 5 min at 10,000× *g* before being frozen at −80 °C for the metabolomics analysis.

### 2.4. Targeted Metabolomics Analysis

We performed a targeted quantitative metabolomic analysis using mass spectrometry (QTRAP 5500, SCIEX, Villebon-sur-Yvette, France) as previously described [10]. In brief, we used the Biocrates^®^ Absolute IDQ p180 kit (Biocrates Life sciences AG, Innsbruck, Austria) which allows us to quantify up to 188 different metabolites: free carnitine (C0), 39 acylcarnitines (C), the sum of hexoses (H1), 21 amino acids, 21 biogenic amines, and 105 lipids. In the kit we used, the lipids are distributed in four different classes: 14 lysophosphatidylcholines (lysoPC), 38 diacyl-phosphatidylcholines (PCaa), 38 acyl-alkyl-phosphatidylcholines (PCae), and 15 sphingomyelins (SM). We used flow-injection analysis with tandem mass spectrometry (FIA-MS/MS) for the analysis of carnitine, acylcarnitines, lipids and hexoses, and we used liquid chromatography (LC) to separate amino acids and biogenic amines.

After thawing, all seminal fluid samples were vortexed and centrifuged at 4 °C for 5 min at 5000× *g*. As per the recommendations of the kit, 10 microliters of each sample were then added to the filter on the upper wells of the 96-well plate for the FIA analysis, and 50 microliters for the LC analysis. Three quality controls (QCs) composed of human plasma samples at three concentration levels (QC1, QC2, and QC3) were added to validate our analysis.

### 2.5. Statistical Analysis

After validation of the kit quality controls, the raw metabolomic data were normalized with UV scaling and mean centering. For each metabolite, the mean concentration was substracted and the difference was divided by the standard error. 

We performed an unsupervised multivariate principal component analysis (PCA), using Simca −P+ v 16.0.1 (Umetrics, Umea, Sweden), in order to visualize the distribution of the metabolomics data and detect the grouping of samples. Hotelling’s T2 plot was used to detect aberrant samples.

We then performed a supervised orthogonal partial least-squares discriminant analysis (OPLS-DA) of the three groups followed by a comparison of each two groups separately. The quality and performance of the models were assessed using different variables: the Q^2^Y(cum) (goodness of the prediction), the R^2^Y(cum) (goodness of the fit), the cross-validation analysis of variance (CV-ANOVA), and the permutation test (evaluation of the overfitting risk).

The significance of the metabolites was assessed using the variable importance in projection (VIP) plot. Only metabolites with a VIP value of at least 1 (VIP ≥ 1) were retained. 

The metabolites found during the different comparisons were all presented as volcano plots using the Simca software −P+ v 16.0.1 (Umetrics).

We depicted the differences between the three groups on a Venn diagram using the software Venny 2.1.0. 

The univariate analyses comparing certain ratios and the sum of metabolites between the groups were performed using the non-parametric Mann–Whitney test on GraphPad Prism v 8.0 (GraphPad Software, San Diego, CA, USA). All tests were considered significant at *p*-value < 0.05.

## 3. Results

### 3.1. Population Characteristics

We included a total of 61 men: 19 in the EO group, out of which 4 had azoospermia and 15 cryptozoospermia, 22 men who had a vasectomy at least 3 months before inclusion in the V group, and 20 men with normal semen parameters according to the WHO classification in the C group (Table S1). 

### 3.2. Metabolomics Signature

We were able to accurately measure 114 metabolites out of the 188 analyzed by the kit. The raw data (concentration of each metabolite in μM in each patient) are presented in the Supplementary Table S2. In total, the following metabolites were correctly measured in the SF of patients, each according to its measuring range: 46 glycerophospholipids out of the 90 analyzed by the kit (24/38 PCaa, 16/38 PCaa and 6/14 LysoPC), all the sphingomyelins (15/15), 20 out of the 21 amino acids, 11 out of the 21 biogenic amines, 21 out of the 39 acylcarnitines, and all the hexoses.

**Figure 1:**
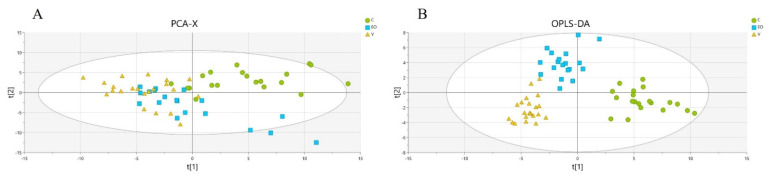
(**A**) Unsupervised PCA score plot of the three populations (EO: blue squares; C: green circles; and V: yellow triangles). (**B**) Supervised OPLS-DA score plot of the three populations. The model constructed with the 114 accurately measured molecules is able to discriminate the three groups. The axes t[1] and t[2] represent the first and second component, respectively, for the PCA, and predictive and orthogonal latent variables for the OPLS-DA.

According to the unsupervised PCA approach, which allows us to visualize the dataset, we did not find any aberrant value or outliers according to Hotelling’s T2 range (Figure 1A). We obtained a discriminating OPLS-DA model that allowed to differentiate the three groups of patients and to confirm that the patients included in the EO group (azoospermia, cryptozoospermia, and severe oligospermia) were homogeneous (Figure 1B). The OPLS-DA model had a good prediction capacity (Q2cum = 70%) and good performances on the permutation test (Q2cy = −0.45) and the CV-ANOVA (*p*-value = 1.33468 × 10^−19^).

We also obtained discriminating OPLS-DA models for the comparisons between each two groups separately (EO vs. C; EO vs. V; V vs. C) (Table 1). These models allowed us to determine the metabolomic signature specific to each group.

**Figure 2:**
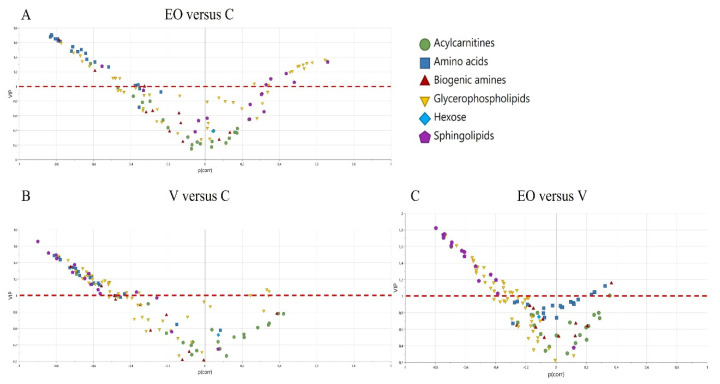
Volcano plot showing the contribution of each metabolite to the corresponding model according to the VIP and the *p*(corr) value. The metabolites with a negative *p*(corr) are decreased, and those with a positive *p*(corr) are increased. Each class of metabolites is represented by a symbol.

The metabolites allowing the distinction between the groups are represented as volcano plots (Figure 2), and the metabolites implicated in these models, two at a time, are the ones with VIP > 1 (list presented in Supplementary Table S3). The metabolites with a negative *p*(corr) are decreased, and those with a positive *p*(corr) are increased.

**Figure 3:**
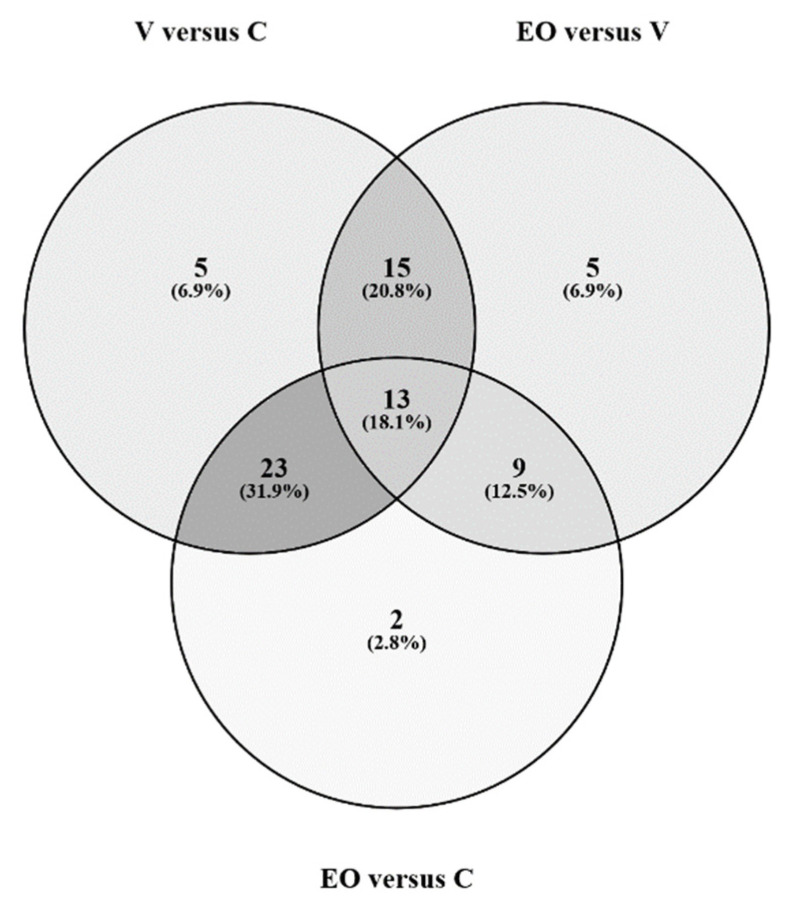
Venn diagram representing the signature metabolites of the three patient groups following pairwise comparison. This diagram was made using: https://bioinfogp.cnb.csic.es/tools/venny/ accessed on 5 December 2022.

All the results are globally represented in the Venn diagram (Figure 3), which shows the 23 metabolites distinguishing the C group (mainly 13 amino acids), the 15 metabolites distinguishing the V group (mainly sphingomyelins (*n* = 7) and free carnitine (C0)), and the 9 metabolites distinguishing the EO group. The complete list of metabolites is presented as supplementary data (Table S4).

**Figure 4:**
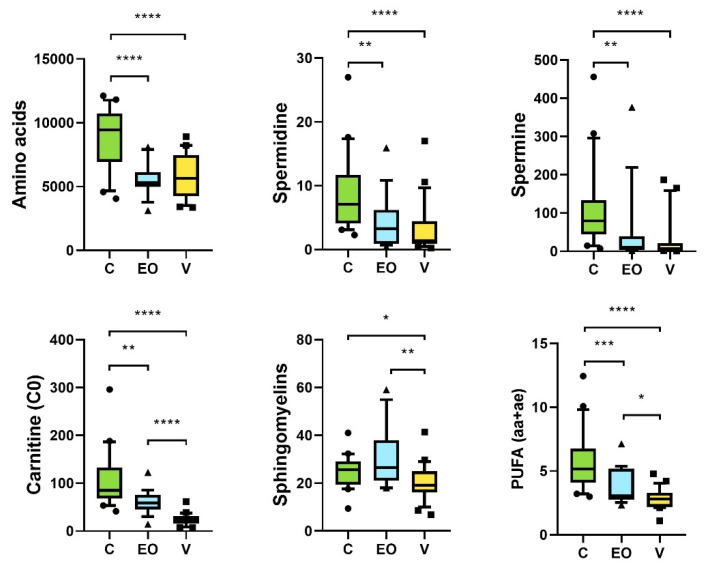
Box plot of the relevant sums in the three groups of patients. The boxes are delimited by the 10th and 90th percentile. The line drawn inside the box represents the median value. The lines on either side of the boxes represent the variation in the data and the individual points (dots, squares, or triangles) represent outliers. *Y* axis represents the concentration of each metabolite in μM. *p*-values were obtained using the non-parametric Mann–Whitney test. *: significant *p*-value < 0.05; ** significant *p*-value < 0.01; *** significant *p*-value < 0.001and **** significant *p*-value < 0.0001.

Figure 4 represents all the metabolites and the groups of metabolites allowing to distinguish the three groups.

The amino acid level and the polyamines (spermine and spermidine) were significantly higher in the C group compared with the other two groups (Figure 4). The sum of polyunsaturated fatty acids (PUFA) and free carnitine progressively decreased between the three groups: they were significantly highest in the C group and lowest in the V group (Figure 4). The sphingomyelins were significantly lower in the V group compared with the C and EO groups (Figure 4).

## 4. Discussion

The seminal fluid is made up of a mixture of secretions from the epididymis, prostate, and seminal vesicles, and reflects the exchanges between the genital tract and the sperm cells. It is essential for the survival of the sperm cells, since it contributes to their nutrition, maturation, and protection from their fertilizing capacity (decapacitation). The exchanges between the sperm cells and the SF occur via exosomes, which represent approximately 3% of its proteins, making it among the richest biologic fluids in exosomes. The exosomes are mainly proteasomes and epididymosomes [18], the latter being secretion vesicles of the epididymal epithelium that are essential for the acquisition of the fertilizing capacity of the sperm cells. They are rich in sphingomyelins and lipids, but contain also RNAs, proteins, and several metabolites such as amino acids [19,20].

In order to analyze the SF, we used a metabolomics approach targeting 188 metabolites with the objective of finding a specific signature that would differentiate men with EO from men with normal semen parameters. We also included a group of vasectomized men in order to have a more detailed understanding of the structures and mechanisms implicated. The signature we found is comprised mainly of amino acids (AA), polyunsaturated fatty acids (PUFA), free carnitine (C0), spermine, and spermidine.

We found a decrease in AA in the EO and V groups when compared with controls. Besides being essential components of proteins, AA play several important roles. It is well known that, in humans, they are abundant in the SF and that they play an essential part in reproduction [21]. On the other hand, in animals, supplementation with certain AA has been shown to improve sperm quality, the fertilizing capacity of sperm cells, and their resistance to cryopreservation [22,23,24]. In men, the administration of AA with antioxidant properties helps to maintain sperm DNA integrity, and the functional parameters of the sperm cells during the cryopreservation process [25].

In infertile men, the levels and composition of AA in the SF seem to be altered. Indeed, studies have shown a global decrease in AA in the semen of azoospermic men [26]. More recently, mass spectrometry studies analyzing the AA content of the SF have revealed an important imbalance in men with asthenospermia [21,27] and oligospermia [21], with a significant decrease in their levels compared with controls. Likewise, metabolomics studies have shown several modifications in the expression profile of AA in the SF according to the sperm quality or the patients’ fertility (metabolism of branched AA) [14], asthenospermia [28], oligoasthenoteratospermia (OATS) [10,12,15], and unexplained infertility [11,29,30,31]. In general, the majority of studies make the case for a decrease in the concentration of AA in the SF in cases of infertility, but there is no consensus on the expression profiles of AA that could be specific to certain sperm alterations, mainly because of the large heterogeneity in the profiles assessed. Our study has focused on EO, a very well-defined and severe phenotype of sperm alteration, and we found a significant decrease in the concentration of the majority of the AA compared with men with normal semen parameters. The presence of sperm cells in the ejaculate does not seem to modify the content in AA of the SF, and a glandular origin of these AAs has been proposed [26]. The fact that the concentration of AA was also lower in vasectomized men (V group) than in controls makes the case for an epididymal origin of the AA. Furthermore, the fact that they are likewise decreased in men with EO suggests an alteration in the “epididymal production” in men with secretory problems.

Our study has also found a significant and progressive decrease in the concentration of PUFA between the C, EO, and V groups, respectively. Lipids are the major components of the sperm membranes, which are mainly comprised of cholesterol and phospholipids that carry saturated and polyunsaturated fatty acids, distributed in an asymmetrical manner. The lipid composition of the spermatic membrane varies across the different developmental stages of sperm cells. During spermatogenesis, there is an integration of PUFA in the membrane via the transformation of essential nutritional fatty acids by elongation and desaturation steps in the germinal cells [32]. During epididymal maturation, there is an increase in the unsaturation level of the fatty acids [33], especially with the incorporation of Docosahexaenoic acid (DHA), which is the main PUFA of the spermatic membrane [34]. The addition of cholesterol during maturation and the upkeep of an asymmetrical distribution of phospholipids allow to ensure a certain membrane stability during periods of stasis and ejaculation [35]. In the female genital tract, and after the cholesterol efflux, they allow increases in the membrane fluidity, which is crucial for the fusion of the sperm and oocyte membranes [35,36].

The link between lipids and male fertility has been proven by several studies analyzing the plasmatic membrane and/or the seminal fluid. The DHA concentration in sperm has been correlated to morphology, motility, and sperm concentration [34,37,38,39]. Many studies have also shown a decrease in the PUFA concentration in the sperm cell membranes in men with asthenospermia [38,40,41,42] and oligospermia [39].

On the other hand, there is a correlation between the lipid composition of the sperm membrane and that of the seminal plasma [43]. Several metabolomics studies have shown an increase in the level of saturated fatty acids [28,44], as well as a decrease in the PUFA levels [34,38] in the seminal plasma of men with asthenospermia, a finding also noted in men with oligoasthenospermia [10]. Finally, it has been proposed that the sperm and the seminal fluid composition in fatty acids could be predictive markers of the success of the sperm freezing procedure [45]. The exact mechanisms of the epididymal rearrangement of the lipid composition of the sperm membranes are not yet fully understood, but they seem to involve the epididymosomes [46]. Indeed, studies have described a variability in the lipid composition of the epididymosomes in the epididymis itself, with the presence at the level of the head of vesicles rich in PUFA and at the level of the tail of vesicles rich in cholesterol and phospholipids [46]. In our study, the decrease in PUFA in the V group is an argument in favor of their epididymosomal origin. The concomitant decrease in sphingomyelins, which are important components of epididymosomes, further supports this hypothesis. Likewise, the decrease in PUFA in the EO group suggests an impairment of the epididymosomal function.

We have also found a progressive decrease in free carnitine (C0) between the control, EO, and V groups. C0 is well known to be primarily of epididymal secretion [47] and has been used as a seminal biochemistry marker. Studies have reported a decrease in free carnitine in infertile men, more specifically men with severe OA [10], and found a correlation between the concentration of C0 and several sperm parameters [48,49].

Finally, we have observed a decrease in several polyamines, such as spermine and spermidine, in the EO and V groups, compared with the controls. These polyamines, which are derived from arginine, play several roles in spermatogenesis and act on cellular proliferation [50]. Spermine also has a role in sperm cell decapacitation [51] and has antioxidant activity that neutralizes free radicals [52]. Polyamines are known to be of prostatic origin [53] but studies have also reported a testicular secretion by Sertoli and Leydig cells [54]. Other studies have indirectly confirmed that finding by showing an alteration of the polyamines concentration in men following vasectomies [55]. Metabolomics studies of the seminal fluid have also shown decreased concentrations of spermine in men with OATS [10,15] and asthenospermia [14].

Surprisingly, we noticed a great similarity between the signature of EO and vasectomized men. That overlapping raises the question whether there is also an epididymal damage associated to the testicular dysfunction (anomalies of spermatogenesis). This double dysfunction has two major consequences. The first is that it highlights the difficulty of finding seminal fluid biomarkers that could help distinguish excretory (non-testicular) from secretory (testicular and non-testicular) azoospermia. The second is that it could constitute an argument in favor of the hypothesis of the testicular dysgenesis syndrome, which states that, in many cases, exposure to environmental toxins, such as endocrine disruptors, and genetic factors can cause an alteration of the spermatogenesis but also malformations of the genital tracts, thus leading to the development of azoospermia and oligospermia [56,57].

## 5. Conclusions

In the current study, we have found a specific signature characterizing extreme oligospermia that includes certain metabolites already linked to infertility in previous studies. Globally, all these metabolites are decreased in men with EO when compared with controls, which rules out a signature linked to the consumption of metabolites by sperm cells. Indeed, the absence or the very low numbers of sperm cells could have led to a non-consumption of metabolites, which would have had higher concentrations than in men with normal semen parameters. This is why this signature most likely reflects a production problem, linked to a dysregulation of the functions and secretions of the genital tract.

## Figures and Tables

**Table 1:** Evaluation of the predictive capacity of different supervised models (OPLS-DA) comparing the three groups (EAS, V, and C). All parameters indicate models with good predictive capacities and low level of overfitting.

Comparisons	Q2Y	Q2(cum)	Q2(cum) Permutations Test	*p*-Value (CV-ANOVA)
V vs. C	0.96	0.87	−0.61	5.67084 × 10^−14^
V vs. EO	0.94	0.76	−0.62	7.38401 × 10^−8^
EO vs. C	0.84	0.75	−0.53	5.97088 × 10^−10^

## Data Availability

The data presented in this study are available in the main article and the supplementary materials.

## Supplementary Materials

The following supporting information can be downloaded at: https://www.mdpi.com/article/10.3390/metabo12121266/s1. Table S1: Characteristics of participant. Table S2: raw data table (attached to the file). Table S3: The relevant metabolites characterizing the multivariate signature of the three groups compared two by two. Molecules were sorted by decreasing VIP value. For each metabolite, the table shows the family, the *p*(corr) value, and the VIP value. Table S4: Metabolites of the Venn diagram.
