# Supplementary material for: A Metabolomic Profile of Seminal Fluid in Extremely Severe Oligozoopermia Suggesting an Epididymal Involvement
Source: Metabolites. 2022 Dec 15;12(12):1266. doi: 10.3390/metabo12121266 (PMC9783920; doi:10.3390/metabo12121266)
Supplement: Supplementary file 1 [file metabolites-12-01266-s001.zip › Supplementary tables.pdf]

Supplementary tables:

Table S1: Characteristics of participants. The non-parametric Mann-Whitney test or the parametric t-test was used for quantitative variables and the chi-squared ($\chi 2$) test for qualitative variables with GraphPad Prism, v.8.0 (GraphPad Software, San Diego, CA, USA). Differences were considered significant at p-value < 0.05.

		**Control group** n = 20	**EO group** n = 19	**V group** n = 22	***p*-value**
**Age (years)**		35.6 ± 6.4	33.5 ± 6.4	41.8 ± 6.4	C vs EO : 0.32<br>C vs V : 0.003<br>EO vs V : 0.0002
**Smoking status**	**Non smoker**	7	13	0	C vs EO : 0.1
	**Smoker**	7	4	0	
	**Information missing**	6	2	22	
**Sperm concentration (10^6 / ml)**		383 ± 220.3	0.3 ± 0.3	0 ± 0	The 3 groups < 0.0001
**Genetic**	**Klinefelter**	0	2	0	C vs EO : 0.18
	**AZF deletion**	0	1	0	
	**None**	20	16	0	
	**Information missing**	0	0	22	
**Urological history**	**Cryptorchidism**	0	6	0	C vs EO < 0.0001
	**Hypospadias**	0	1	0	
	**Testicular hypotrophy**	0	13	0	
	**Other**	0	3	0	
	**None**	20	6	0	
	**Information missing**	0	0	22	

Table S3: The relevant metabolites characterizing the multivariate signature of the three groups compared two by two. Molecules were sorted by decreasing VIP value. For each metabolite, the table shows the family, the *p*(corr) value, and the VIP value.

**C versus V (increased or decreased in V)**				**EO versus V (increased or decreased in EO)**				**EO versus C (increased or decreased in EO)**			
**Identification**		**Multivariate analysis**		**Identification**		**Multivariate analysis**		**Identification**		**Multivariate analysis**	
**Molecules**	**Family**	**P(corr) value**	**VIP value**	**Molecules**	**Family**	**P(corr) value**	**VIP value**	**Molecules**	**Family**	**P(corr) value**	**VIP value**
SM C18:1	Sphingolipids	-0.899	1.656	SM (OH) C22:1	Sphingolipids	-0.795	1.825	Lysine	Amino acids	-0.825	1.707
SM (OH) C22:1	Sphingolipids	-0.840	1.518	SM C24:1	Sphingolipids	-0.739	1.749	Aspartate	Amino acids	-0.822	1.694
SM C16:1	Sphingolipids	-0.800	1.492	SM (OH) C24:1	Sphingolipids	-0.747	1.742	Arginine	Amino acids	-0.831	1.678
Lysine	Amino acids	-0.811	1.487	PC aa C36:1	Glycerophospholipids	-0.731	1.738	Glutamate	Amino acids	-0.804	1.653
PC aa C32:0	Glycerophospholipids	-0.787	1.485	SM C24:0	Sphingolipids	-0.745	1.707	Glycine	Amino acids	-0.792	1.651
Aspartate	Amino acids	-0.787	1.463	SM (OH) C22:2	Sphingolipids	-0.690	1.652	Ac-Ornithine	Biogenic amines	-0.785	1.634
SM (OH) C22:2	Sphingolipids	-0.797	1.449	PC aa C32:0	Glycerophospholipids	-0.660	1.616	Threonine	Amino acids	-0.789	1.624

Glutamate	Amino acids	-0.778	1.434	SM C26:1	Sphingolipids	-0.695	1.610	Serine	Amino acids	-0.778	1.621
PC aa C38:6	Glycerophospholipids	-0.736	1.431	C0	Acylcarnitines	-0.694	1.597	PC aa C38:6	Glycerophospholipids	-0.773	1.595
SM C18:0	Sphingolipids	-0.700	1.376	SM C18:0	Sphingolipids	-0.624	1.548	Alanine	Amino acids	-0.710	1.544
Ac-Ornithine	Biogenic amines	-0.721	1.351	SM (OH) C16:1	Sphingolipids	-0.606	1.534	Methionine	Amino acids	-0.665	1.504
Glycine	Amino acids	-0.708	1.342	SM C18:1	Sphingolipids	-0.606	1.482	Asparagine	Amino acids	-0.717	1.484
Arginine	Amino acids	-0.728	1.339	PC ae C36:1	Glycerophospholipids	-0.557	1.468	Glutamine	Amino acids	-0.684	1.475
Alanine	Amino acids	-0.693	1.329	PC aa C30:0	Glycerophospholipids	-0.547	1.437	PC aa C40:6	Glycerophospholipids	-0.706	1.464
PC aa C40:6	Glycerophospholipids	-0.657	1.329	PC aa C38:3	Glycerophospholipids	-0.548	1.425	Tryptophan	Amino acids	-0.641	1.455
PC ae C40:6	Glycerophospholipids	-0.694	1.300	SM C16:1	Sphingolipids	-0.532	1.362	PC ae C40:6	Glycerophospholipids	-0.670	1.422
C5:1	Acylcarnitines	-0.693	1.292	PC ae C34:0	Glycerophospholipids	-0.519	1.354	Proline	Amino acids	-0.634	1.370
Proline	Amino acids	-0.681	1.292	PC ae C34:1	Glycerophospholipids	-0.479	1.349	lysoPC a C16:1	Glycerophospholipids	0.646	1.367
PC aa C38:3	Glycerophospholipids	-0.638	1.288	PC ae C32:1	Glycerophospholipids	-0.529	1.314	PC aa C36:1	Glycerophospholipids	0.659	1.351

SM C24:0	Sphingolipids	-0.711	1.283	PC aa C40:4	Glycerophospholipids	-0.530	1.286	PC ae C38:6	Glycerophospholipids	-0.660	1.346
SM (OH) C16:1	Sphingolipids	-0.623	1.269	SM C16:0	Sphingolipids	-0.433	1.260	Histidine	Amino acids	-0.591	1.334
Methionine	Amino acids	-0.626	1.262	PC aa C38:4	Glycerophospholipids	-0.478	1.246	SM C24:1	Sphingolipids	0.660	1.334
C0	Acylcarnitines	-0.691	1.261	SM (OH) C14:1	Sphingolipids	-0.399	1.197	lysoPC a C18:1	Glycerophospholipids	0.576	1.331
PC ae C38:6	Glycerophospholipids	-0.608	1.247	SM C26:0	Sphingolipids	-0.512	1.184	lysoPC a C17:0	Glycerophospholipids	0.548	1.316
Asparagine	Amino acids	-0.674	1.244	PC aa C36:2	Glycerophospholipids	-0.354	1.180	C5:1	Acylcarnitines	-0.615	1.312
PC aa C36:1	Glycerophospholipids	-0.631	1.242	lysoPC a C18:2	Glycerophospholipids	-0.470	1.169	lysoPC a C16:0	Glycerophospholipids	0.528	1.297
Tryptophan	Amino acids	-0.608	1.238	PC aa C36:4	Glycerophospholipids	-0.414	1.164	PC aa C34:1	Glycerophospholipids	0.506	1.281
PC aa C36:6	Glycerophospholipids	-0.643	1.231	PC aa C34:1	Glycerophospholipids	-0.346	1.158	lysoPC a C18:0	Glycerophospholipids	0.499	1.279
Tyrosine	Amino acids	-0.618	1.216	Ac-Ornithine	Biogenic amines	0.366	1.157	PC aa C36:6	Glycerophospholipids	-0.620	1.279
PC aa C42:4	Glycerophospholipids	-0.659	1.212	Isoleucine	Amino acids	0.324	1.124	SM C18:1	Sphingolipids	-0.553	1.277
SM C26:1	Sphingolipids	-0.646	1.208	PC aa C36:3	Glycerophospholipids	-0.350	1.106	Leucine	Amino acids	-0.518	1.264

PC aa C40:5	Glycerophospholipids	-0.604	1.201	PC aa C38:6	Glycerophospholipids	-0.433	1.100	PC ae C36:1	Glycerophospholipids	0.533	1.244
PC aa C30:0	Glycerophospholipids	-0.538	1.183	PC aa C34:2	Glycerophospholipids	-0.260	1.070	alpha-AAA	Biogenic amines	-0.591	1.214
PC aa C38:5	Glycerophospholipids	-0.553	1.166	PC ae C40:2	Glycerophospholipids	-0.410	1.065	PC aa C28:1	Glycerophospholipids	0.468	1.207
PC aa C36:3	Glycerophospholipids	-0.512	1.164	PC ae C34:2	Glycerophospholipids	-0.304	1.052	PC ae C34:0	Glycerophospholipids	0.458	1.196
PC aa C40:3	Glycerophospholipids	-0.625	1.162	Leucine	Amino acids	0.256	1.052	SM C16:0	Sphingolipids	0.437	1.179
PC aa C38:4	Glycerophospholipids	-0.518	1.158	lysoPC a C16:1	Glycerophospholipids	-0.339	1.050	PC aa C36:3	Glycerophospholipids	-0.484	1.120
Serine	Amino acids	-0.574	1.153	Glutamine	Amino acids	0.253	1.048	PC aa C38:3	Glycerophospholipids	-0.495	1.115
Threonine	Amino acids	-0.596	1.151	PC aa C28:1	Glycerophospholipids	-0.289	1.042	PC aa C40:5	Glycerophospholipids	-0.481	1.113
PC aa C40:4	Glycerophospholipids	-0.590	1.140	SM C20:2	Sphingolipids	-0.387	1.033	PC aa C38:5	Glycerophospholipids	-0.459	1.113
Ornithine	Amino acids	-0.610	1.140	Serine	Amino acids	0.234	1.021	SM (OH) C14:1	Sphingolipids	0.354	1.107
SM (OH) C24:1	Sphingolipids	-0.613	1.136	C5-DC (C6-OH)	Acylcarnitines	0.354	1.006	SM C24:0	Sphingolipids	0.481	1.056
PC aa C34:2	Glycerophospholipids	-0.518	1.136					PC aa C36:2	Glycerophospholipids	0.265	1.041

Histidine	Amino acids	-0.567	1.131
Spermidine	Biogenic amines	-0.556	1.113
SM C24:1	Sphingolipids	-0.575	1.073
lysoPC a C18:1	Glycerophospholipids	0.333	1.070
lysoPC a C16:0	Glycerophospholipids	0.346	1.053
PC ae C32:1	Glycerophospholipids	-0.512	1.049
SM (OH) C14:1	Sphingolipids	-0.368	1.044
PC aa C36:4	Glycerophospholipids	-0.421	1.040
PC aa C36:2	Glycerophospholipids	-0.354	1.035
PC ae C34:1	Glycerophospholipids	-0.467	1.026
SM C26:0	Sphingolipids	-0.563	1.023
Valine	Amino acids	-0.433	1.020
PC aa C30:0	Glycerophospholipids	0.342	1.025
SM (OH) C16:1	Sphingolipids	0.328	1.024
Valine	Amino acids	-0.358	1.021
Isoleucine	Amino acids	-0.375	1.013

lysoPC a C18:0	Glycerophospholipids	0.245	1.004

Table S4: Metabolites of the Venn diagram

	Common to all three comparisons	C versus (V-EO)	V versus (C-EO)	EO versus (C-V)	C versus V	EO versus V	EO versus C
Class of metabolites	13 metabolites	23 metabolites	15 metabolites	9 metabolites	5 metabolites	5 metabolites	2 metabolites
Amino acids	Ser	Lys Met<br>Asp Asn<br>Glu Trp<br>Gly Thr<br>Arg His<br>Ala Val<br>Pro		Gln<br>Ile<br>Leu	Orn<br>Tyr		
Biogenic amines	Ac-Orn				Spermidine		alpha-AAA
Acylcarnitines		C5:1	C0			C5-DC (C6-OH)	
Glycerophospholipids	PC aa C30:0	PC aa C36:6	PC aa C32:0	PC aa C28:1	PC aa C40:3	PC ae C34:2	lysoPC a C17:0

	PC aa C36:1<br>PC aa C36:2<br>PC aa C36:3<br>PC aa C38:3<br>PC aa C38:6	PC aa C40:5<br>PC aa C38:5<br>PC ae C38:6<br>PC aa C40:6<br>PC ae C40:6<br><br>lysoPC a C18:1<br>lysoPC a C16:0<br>lysoPC a C18:0	PC aa C34:2<br>PC aa C36:4<br>PC aa C38:4<br>PC aa C40:4<br>PC ae C32:1<br>PC ae C34:1	PC aa C34:1<br>PC ae C34:0<br>PC ae C36:1<br><br>lysoPC a C16:1	PC aa C42:4	PC ae C40:2<br><br>lysoPC a C18:2	
Sphingomyelins	SM (OH) C14:1<br>SM (OH) C16:1<br>SM C18:1<br>SM C24:0<br>SM C24:1		SM (OH) C22:1<br>SM (OH) C22:2<br>SM (OH) C24:1<br>SM C16:1<br>SM C18:0<br>SM C26:0<br>SM C26:1	SM C16:0		SM C20:2	
